# Prognostic Value of Fibrosis 4 (FIB-4) Index in Sepsis Patients

**DOI:** 10.3390/jpm14050531

**Published:** 2024-05-16

**Authors:** Tuna Albayrak, Beyza Yuksel

**Affiliations:** 1 Department of Anesthesiology and Reanimation, Giresun University Faculty of Medicine, Giresun 28200, Türkiye; 2 Giresun Training and Research Hospital, Internal Medicine Intensive Care Unit, Giresun 28200, Türkiye; beyzcan@gmail.com

**Keywords:** sepsis prognosis, FIB-4 index, intensive care units (ICUs), early mortality prediction, risk stratification

## Abstract

Background: Sepsis remains a major health challenge worldwide, characterized by a dysregulated host response to infection, leading to high mortality and morbidity in intensive care units (ICUs). The Fibrosis 4 (FIB-4) index, originally developed to assess liver fibrosis in hepatitis C patients, has recently been explored for its potential prognostic value in sepsis patients. Method: this study retrospectively analyzed 309 sepsis patients admitted to the Internal Medicine and An-aesthesia ICUs between 12 December 2021 and 15 December 2023 to investigate the relationship between FIB-4 levels, the Acute Physiology and Chronic Health Evaluation (APACHE), the Sequential Organ Failure Assessment (SOFA), and clinical outcomes. Results: This study found that higher FIB-4 measurements were statistically significantly associated with increased 28-day mortality, with a cut-off value of 4.9, providing a sensitivity of 54.92% and specificity of 74.25%. Logistic regression analysis indicated that elevated FIB-4 levels were a significant predictor of early mortality, suggesting that the FIB-4 index could serve as a valuable prognostic tool in assessing the severity and prognosis of sepsis patients. Conclusions: by elucidating the potential role of the FIB-4 index in sepsis prognosis, this study contributes to the ongoing efforts to improve risk stratification and enhance patient care in sepsis management.

## 1. Introduction

Sepsis, a life-threatening condition characterized by a dysregulated host response to infection, continues to be a significant challenge in critical care and emergency medicine [1]. Despite advancements in understanding its pathophysiology and improvements in supportive care, sepsis and its severe manifestations, such as septic shock, remain leading causes of mortality and morbidity in intensive care units (ICUs) worldwide [2]. The complexity of sepsis, marked by its heterogeneous presentation and progression, necessitates the identification and utilization of reliable prognostic markers to guide clinical decision making and improve patient outcomes [3].

Here is a concise and accurate response to the question, drawing from the given search results.

Severity scoring systems and prognostic models are important tools used in intensive care units (ICUs) to assess the severity of illness and predict patient outcomes. These systems enable the comparative audits and evaluative research of ICUs [1,4].

The ideal severity scoring system should include easily measured, objective, and reproducible parameters collected during routine patient management. Some of the most commonly used severity scoring systems include the Acute Physiology and Chronic Health Evaluation (APACHE), Simplified Acute Physiology Score (SAPS), and Sequential Organ Failure Assessment (SOFA). These models aim to stratify patients based on the severity of illness and predict outcomes like in-hospital mortality. They have been extensively validated, predominantly in high-income countries, and generally demonstrate good discrimination and calibration. However, the performance of these models may be limited in low- and middle-income country settings due to differences in the case-mix, availability of predictor variables, and data collection challenges. Efforts are ongoing to develop and validate context-specific prognostic models for these settings [5,6].

Overall, severity scoring systems and prognostic models are essential tools for ICU care, but their appropriate application and interpretation is crucial to avoid misuse and ensure optimal utility in guiding patient management and resource allocation [1]. 

The Fibrosis 4 (FIB-4) index, originally developed to non-invasively assess liver fibrosis in patients with hepatitis C, has emerged as a potential prognostic marker in various clinical settings beyond liver disease [7]. The FIB-4 index is calculated based on readily available laboratory parameters: age, aspartate aminotransferase (AST), alanine aminotransferase (ALT), and platelet count. This simplicity and non-invasiveness make the FIB-4 index an attractive tool for clinical use [8]. Recent studies have suggested that the FIB-4 index may reflect not only liver fibrosis, but also systemic inflammation and organ dysfunction, which are central to the pathophysiology of sepsis [9]. The prognostic value of the FIB-4 index in sepsis patients remains an area of active investigation. The preliminary evidence indicates that elevated FIB-4 levels may be associated with worse outcomes in sepsis, including increased mortality, longer ICU stays, and higher rates of organ dysfunction. These associations are thought to arise from the FIB-4 index’s ability to capture the extent of systemic inflammation and its impact on multiple organ systems, including the liver [10]. Given the liver’s pivotal role in modulating immune responses and its susceptibility to damage in the context of sepsis, the FIB-4 index could provide valuable insights into the severity and prognosis of septic patients. 

This study aims to comprehensively evaluate the prognostic value of the FIB-4 index in patients with sepsis admitted to the ICU. By elucidating the relationship between FIB-4 levels and clinical outcomes in sepsis, we hope to contribute to the ongoing efforts to improve risk stratification, guide therapeutic interventions, and ultimately enhance the care of patients facing this formidable challenge.

## 2. Materials and Methods

### 2.1. Study Design and Population

This retrospective cohort study included patients who were admitted to the Internal Medicine Intensive Care Unit and Anesthesia Intensive Care Unit at Giresun Training and Research Hospital with a diagnosis of sepsis between 12 December 2021 and 15 December 2023.

Ethics Consideration: the study design conformed to the Declaration of Helsinki and was approved by Giresun Training and Research Hospital Ethics Committee on 25 December 2023 with the number E-53593568-771-232700672.

Inclusion Criteria: patients diagnosed with sepsis upon admission or within the first 48 h of hospitalization (community-acquired sepsis) or developing sepsis 48 h after hospitalization (nosocomial sepsis), patients for whom complete medical records are available, including laboratory and clinical data necessary for the calculation of the FIB-4 index and APACHE II score.

Exclusion Criteria: atients under 18 years of age, patients with incomplete medical records or missing data necessary for the analysis, and patients who were discharged or transferred to another facility within 48 h of admission.

### 2.2. Data Collection

Data were collected retrospectively from patient medical records. The following information was recorded for each patient: age, gender, body mass index (BMI), comorbidity status, sepsis status, focus of infection, microorganism produced, mean arterial pressure, C-reactive protein (CRP), procalcitonin, lactate, aspartate aminotransferase (AST), alanine aminotransferase (ALT), total and direct bilirubin, urea, creatinine, white blood cell count, neutrophil count, and platelet count. Additionally, severity scores such as the APACHE II score, Sequential Organ Failure Assessment (SOFA) score, inotrope requirement, duration of intensive care unit stay, and discharge or exit status were also recorded.

FIB-4 Index Calculation

FIB-4 = Age(years) × AST (U/L)/Platelet count (×10^9^/L) × ALT^1/2^ (U/L)

### 2.3. Statistical Investigations

The SPSS 26 (Statistical Package for the Social Sciences) program was used for statistical analysis. While evaluating the study data, quantitative variables were shown with mean, standard deviation, median, min, and max values and qualitative variables were shown with descriptive statistical methods such as frequency and percentage. A Shapiro–Wilks test and Aox Plot graphs were used to evaluate the conformity of the data to a normal distribution. A Student’s *t*-test was used for the quantitative evaluations of two groups with normal distribution, and a Mann–Whitney-U test was used for the evaluations of variables that did not show normal distribution according to two groups. 

The Chi-square test and Fisher’s Exact test were used to compare qualitative data. Diagnostic screening tests and ROC analysis were used to determine the cut off of FIB-4 measurements according to mortality. 

Logistic Regression analysis was used for multivariate evaluations of risk factors affecting early mortality. The results were evaluated at 95% confidence interval and significance was evaluated at *p* < 0.05 level.

## 3. Results

This study was conducted at Giresun Training and Research Hospital between 12 December 2021 and 15 December 2023, involving a total of 309 patients, 42.1% of whom were female (*n* = 130) and 57.9% of whom were male (*n* = 179). The ages of the patients ranged from 27 to 98 years, with a mean age of 74.78 ± 13.92 years (Table 1). 

Comorbidities such as hypertension (69.3%; *n* = 214), diabetes mellitus (29.1%; *n* = 90), chronic renal failure (37.9%; *n* = 117), left ventricular hypertrophy (15.5%; *n* = 48), and coronary artery disease (33%; *n* = 102) were observed. Nosocomial infections occurred in 72.8% of cases (*n* = 225), and 34% of participants (*n* = 105) experienced septic shock (Table 2).

The sites of infection were blood in 40.1% (*n* = 124), catheter in 6.8% (*n* = 21), tracheal aspirate culture/sputum in 36.2% (*n* = 112), urine in 30.1% (*n* = 93), wound site in 6.5% *n* = 20), and other in 1.3% (*n* = 4).

Patients were divided into two groups: Survival (*n* = 167) and Non-Survival (*n* = 142).

There were no statistically significant differences between the two groups in terms of gender, the presence of hospital infection, or causative agents (*p* > 0.05). However, the rate of diabetes mellitus was statistically significantly lower (*p* = 0.009) and the rate of septic shock was higher (*p* = 0.001) in non-survivors. Additionally, non-survivors had a lower rate of infection originating from the wound site (*p* = 0.004) (Table 2).

An analysis of age and biochemical measurements did not find any statistically significant differences between the groups in terms of age and creatinine levels (*p* > 0.05). However, non-survivors had statistically significantly higher APACHE II scores (*p* = 0.001), SOFA scores (*p* = 0.001), lactate levels (*p* = 0.001), AST levels (*p* = 0.011), total bilirubin levels (*p* = 0.001), direct bilirubin levels (*p* = 0.001), urea levels (*p* = 0.001), and FIB-4 levels (*p* = 0.001) (Table 3). ROC analysis for FIB-4 established a cut-off value of 4.9, with a sensitivity of 54.92%, specificity of 74.25%, positive predictive value of 64.46%, and negative predictive value of 64.95% (Table 4). The area under the ROC curve was 67.5% with a standard error of 3.1% (Table 4).

**Figure 1:**
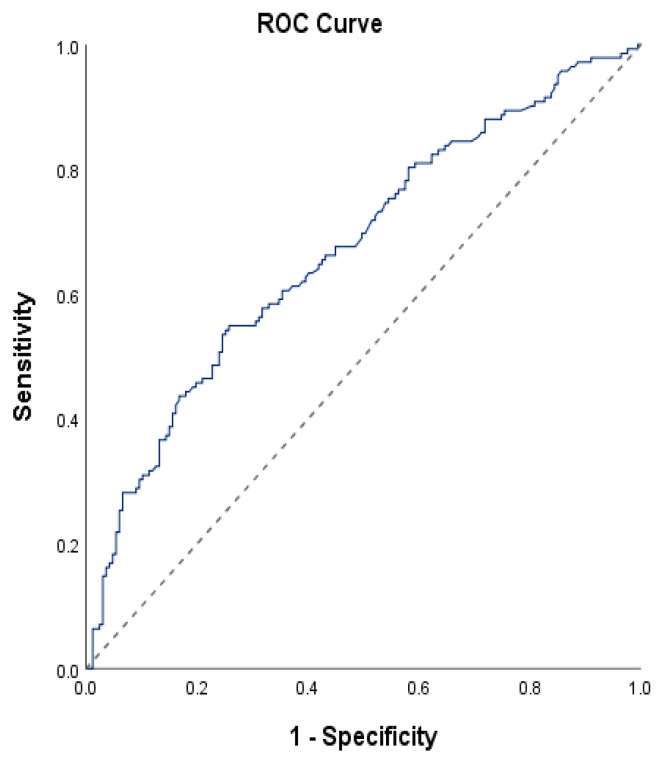
ROC curve of FIB-4 level by mortality.

A significant correlation was found between non-survivors and the cut-off value of 4.9 for FIB-4 (*p* = 0.001); the risk of non-survivors increased 3.52-fold in patients with a FIB-4 level of 4.9 and above. The odds ratio for the FIB-4 measurement was 3.515 (95% CI: 2.177–5.675) (Table 4, Figure 1).

Logistic regression analysis evaluated the effects of diabetes mellitus, septic shock, wound site infection, APACHE II, SOFA, lactate, AST, total and direct bilirubin, urea, and FIB-4 on non-survivors. The model was significant (F = 78.391; *p* = 0.001) and had a good explanatory coefficient of 71.2%. APACHE II, SOFA, direct bilirubin levels, and FIB-4 were independent risk factors for non-survivors (*p* < 0.05). A one-unit increase in the APACHE II scores increased the odds ratio for non-survivors by 1.101 (95% CI: 1.008–1.156). Similarly, a one-unit increase in the SOFA scores increased the odds ratio for non-survivors by 1.122 (95% CI: 1.007–1.251). A one-unit increase in the direct bilirubin levels increased the odds ratio for non-survivors by 1.228 (95% CI: 1.080–1.497). Additionally, a FIB-4 level of 4.9 and above increased the odds ratio for non-survivors by 2.127 (95% CI: 1.237–3.659). Although diabetes mellitus, septic shock, wound site infection, lactate, AST, total bilirubin, and urea were significant in the univariate analyses, they were not significant in the multivariate evaluation (*p* > 0.05) (Table 5).

In conclusion, APACHE II, SOFA, direct bilirubin levels, and FIB-4 ≥ 4.9 are independent risk factors for non-survivors in patients.

## 4. Discussion

In our study, we planned to investigate whether there is a relationship between the severity of sepsis and mortality, the APACHEII score, and the FIB-4 score. 

Our study highlights the prognostic significance of the Fibrosis-4 (FIB-4) index in sepsis patients admitted to the intensive care unit. The retrospective analysis, covering a cohort of 309 patients, revealed a statistically significant association between elevated FIB-4 levels and increased mortality rates. For example, an FIB-4 cut-off value of ≥4.9 had a sensitivity of 54.92% and a specificity of 74.25% for predicting death, with an odds ratio of 3.515. This meant that patients with a FIB-4 level of 4.9 or higher had a 3.52-times higher risk of dying early than those with lower FIB-4 levels.

Furthermore, our analysis found that APACHE II, SOFA, and direct bilirubin levels, along with FIB-4, are independent risk factors for early mortality in sepsis patients. The APACHE II and SOFA scores are well-established prognostic tools in critical care, reflecting the severity of illness and organ dysfunction, respectively. Adding FIB-4 as a separate risk factor to these established scores suggests that FIB-4’s measurement of liver function is very important in determining the outcome of sepsis patients. This is particularly relevant given the liver’s central role in the inflammatory response and its susceptibility to damage during sepsis. 

Patients with conditions like viral hepatitis and non-alcoholic fatty liver disease (NAFLD) often use the Fibrosis-4 (FIB-4) index, a non-invasive scoring system, to evaluate liver fibrosis. The age, platelet count, aspartate aminotransferase (AST), and alanine aminotransferase (ALT) levels are the basis for its calculation [7,11,12]. The FIB-4 index has been found to be a useful tool not only in assessing liver fibrosis, but also in predicting clinical outcomes in various patient populations, including those with sepsis and other critical illnesses [13,14,15,16,17,18].

Previous research has shown a positive correlation between age and FIB-4 values, indicating that older individuals may exhibit higher levels of liver fibrosis [19]. However, in this study, neither age nor creatinine measurements showed statistically significant differences concerning mortality (*p* > 0.05). One reason for this could be that the FIB-4 index can accurately distinguish advanced fibrosis in younger patients, but may have limited diagnostic accuracy in older patients. Thus, an adjustment for age’s impact on the FIB-4 index may be necessary for a more accurate classification in older patients. 

Diabetic patients are particularly vulnerable to liver fibrosis. A cross-sectional study found that 23.8% of diabetic adults had significant liver fibrosis, while 15.4% had advanced liver fibrosis [20]. This vulnerability is largely due to insulin abnormalities. Insulin is essential for the liver’s normal function as it helps regulate glucose uptake. Insulin resistance can lead to hepatic lipid accumulation and disrupted glucose regulation, which can eventually cause liver fibrosis [21]. 

Beyond insulin resistance as a risk factor for liver fibrosis, other contributors include hepatitis, excessive alcohol consumption, and non-alcoholic fatty liver disease (NAFLD). NAFLD is the most common cause of liver fibrosis and is highly prevalent in diabetic patients, especially those with type 2 diabetes (T2DM). Around 55.48% of T2DM patients also have NAFLD. The prevalence of advanced liver fibrosis is notably higher in patients with both T2DM and NAFLD (17.02%) compared to those with only T2DM (4.80%) [22,23].

Furthermore, liver fibrosis can increase the risk of cardiovascular disease (CVD). Therefore, diabetic patients with liver fibrosis may face a significantly higher risk of developing CVD [22,24].

However, the rate of diabetes mellitus was statistically significantly lower (*p* = 0.009) and the rate of septic shock was higher (*p* = 0.001) in non-survivors. However, diabetic patients in this study may have different characteristics compared to the general diabetic population in other studies. Factors such as age, general health status, adherence to treatment and the presence of other comorbidities may affect the results. 

Sepsis is a severe and life-threatening condition that arises when the body’s response to an infection becomes uncontrolled, leading to widespread inflammation and potential organ failure. This condition can progress rapidly, making early recognition and treatment crucial [25].

In septic patients, an elevated FIB-4 score has been associated with poor outcomes, such as increased mortality and the need for renal replacement therapy. The index serves as an independent short-term mortality scoring system, indicating that advanced stages of subclinical hepatic fibrosis can lead to worse outcomes in these patients. This association is also observed in non-septic critically ill patients, suggesting the generalizability of FIB-4 as a prognostic tool in critical care settings [10]. 

External validation studies have shown that FIB-4 is a reliable tool for predicting out- comes in septic patients, with results that are similar to those from primary studies [10]. This supports the use of FIB-4 as a supplementary tool alongside existing prognostic scoring systems to enhance the prediction of clinical outcomes [1].

Beyond liver diseases, FIB-4 has been applied to other clinical scenarios, such as cardiovascular diseases and infections like COVID-19. For instance, it has been used to predict the need for mechanical ventilation in COVID-19 patients, with specific cutoff values providing significant predictive accuracy [9,26].

The liver is a target organ for the SARS-CoV-2 virus and can be affected by the inflammatory response to the infection. Liver injury has been observed in COVID-19 patients. Elevated AST and ALT levels have been linked to poorer outcomes in COVID-19 patients, although the underlying mechanisms remain unclear. Patients with higher FIB-4 scores often have increased liver disease and overall mortality. While severe liver injury and liver failure are uncommon in COVID-19 patients, long-term effects post-infection are still unknown [26]. 

In non-alcoholic fatty liver disease (NAFLD) patients, FIB-4 is associated with liver disease progression and mortality, but may not accurately predict liver-related mortality and morbidity in diabetic NAFLD patients. Beyond liver diseases, FIB-4 has also been linked to all-cause mortality in systemic chronic illnesses such as rheumatoid arthritis, microscopic polyangiitis, and chronic obstructive pulmonary disease [27]. 

A higher FIB-4 index is associated with an increased incidence of renal failure. Therefore, the FIB-4 index may be useful in identifying patients who are at risk not only of liver-related events, but also of renal disease. However, in this study, creatinine measurements of the patients according to mortality did not show a statistically significant difference. 

Studies have demonstrated that FIB-4 outperforms other liver fibrosis indices such as the NAFLD fibrosis score (NFS) and AST to Platelet Ratio Index (APRI). It has been particularly effective in differentiating the stages of liver fibrosis in patients with chronic viral hepatitis and NAFLD. Studies have demonstrated its utility in predicting long-term outcomes such as the hepatocellular carcinoma incidence and mortality in these patient groups [10,28].

The study provided highlights the importance of the Fibrosis-4 (FIB-4) index as an independent predictor of short-term mortality in septic patients. This suggests the potential value of incorporating the FIB-4 index into existing prognostic tools in critical care settings for a more comprehensive assessment of patient outcomes. 

This study comprehensively evaluates the prognostic value of the Fibrosis-4 (FIB-4) index in sepsis patients admitted to the intensive care unit. It examines the association between elevated FIB-4 levels and not only mortality rates, but also other important clinical outcomes such as the need for mechanical ventilation and renal replacement therapy. 

There is limited research on the prognostic value of the FIB-4 index in the context of sepsis. This study adds to the growing body of evidence supporting the use of FIB-4 as a prognostic marker in critical illness, beyond its traditional application in chronic liver dis- eases. However, this is the first study in the literature comparing the FIB-4 index with the traditionally used SOFA scoring system and the APACHEE scoring system. In this study, similar to the study of Zhu, X. et al. [10], an increase in the FIB-4 index is associated with unfavorable results. However, in our study, in addition to the FIB-4 index, the SOFA score and APACHE scoring system were also studied and a comparison was also made between them.

This study emphasizes that elevated levels of the FIB-4 index are associated with an increased mortality risk in septic patients, indicating a 3.52-times higher risk of early mortality for patients with a FIB-4 level of 4.9 and above. The FIB-4 index, initially designed to assess liver fibrosis in chronic liver diseases, has potential as a prognostic marker in sepsis, alongside established prognostic tools like the APACHE II and SOFA scores. Elevated FIB-4 levels were associated with adverse outcomes in septic patients, including an increased need for invasive mechanical ventilation and renal replacement therapy [6]. 

### Study Limitations

This study’s retrospective design presents potential limitations, particularly regarding the accuracy and completeness of the data obtained. Data collected in the past may lead to missing important information. Furthermore, the number and diversity of patient samples utilized could impact the generalizability of the findings. Studies involving larger and more diverse populations might offer more generalized results. Additionally, the analysis of risk factors in this study may be limited, potentially overlooking other factors associated with sepsis. External validation studies are necessary to confirm the findings, and results from similar studies involving different populations could enhance the reliability of this study’s conclusions. Given the heterogeneous nature of sepsis cases, this study’s results may not be universally applicable.

## 5. Conclusions

This study emphasizes the multifactorial nature of sepsis prognosis and the need for integrating clinical, demographic, and laboratory parameters to guide risk stratification and management decisions. Using the FIB-4 index, clinicians may be able to improve out- comes and reduce mortality in septic patients. Future prospective studies are necessary to validate these findings and explore the clinical utility of incorporating the FIB-4 index into sepsis management protocols.

## Figures and Tables

**Table 1:** Distribution of Sociodemographic Characteristics.

		*n* (%)
Gender	Female	130 (42.1)
	Male	179 (57.9)
Age	Mean ± Ss	74.78 ± 13.92
	Median (Min–Max)	77 (27–98)
º Comorbidity	HT	214 (69.3)
	DM	90 (29.1)
	CKD	117 (37.9)
	CVD	48 (15.5)
	CAD	102 (33.0)
Nasocomial infection	No	84 (27.2)
	Yes	225 (72.8)
Septic shock	No	204 (66.0)
	There is	105 (34.0)
º Breeding place	Blood	124 (40.1)
	Catheter	21 (6.8)
	Tracheal aspirate culture/Phlegm	112 (36.2)
	Urine	93 (30.1)
	Wound site	20 (6.5)
	Other (pleural fluid, peritoneum, etc.)	4 (1.3)
Effective	Acineto	20 (6.5)
	*Klebsiella*	44 (14.2)
	*Pseudomonas*	46 (14.9)
	*E. coli*	96 (31.1)
	*Enterococcus*	34 (11.0)
	MRSA	10 (3.2)
	MSSA	15 (4.9)
	Candida	17 (5.5)
	Other	33 (10.7)
28-day mortality	Survival	167 (54.0)
	Non-survival	142 (46.0)

º More than one option is selected. MSSA: methicillin-sensitive Staphylococcus aureus, MRSA: Methicillin-Resistant Staphylococcus aureus, HT: Hypertension, DM: Diabetes Mellitus, CVD: Cerebrovascular disease, CAD: Coroner artery disease.

**Table 2:** Evaluation of Descriptive Characteristics According to Mortality.

		28-Day Mortality		*p*
		Survival (*n* = 167)	Non-Survival (*n* = 142)	
Gender	Woman	67 (40.1)	63 (44.4)	^a^ 0.451
	Male	100 (59.9)	79 (55.6)	
Comorbidity	HT	119 (71.3)	95 (66.9)	^a^ 0.408
	DM	59 (35.3)	31 (21.8)	^a^ 0.009 **
	CKD	66 (39.5)	51 (35.9)	^a^ 0.515
	CVD	32 (19.2)	16 (11.3)	^a^ 0.056
	CAD	60 (35.9)	42 (29.6)	^a^ 0.237
Nasocomial	No			^a^ 0.101
		39 (23.4)	45 (31.7)	
	Yes	128 (76.6)	97 (68.3)	
Septic shock	No	93 (55.7)	111 (78.2)	^a^ 0.001 **
	Yes	74 (44.3)	31 (21.8)	
	Place of reproduction Blood			^a^ 0.639
		0.65 (38.9)	59 (41.5)	
	Catheter	11 (6.6)	10 (7.0)	^a^ 0.874
	TAC/Phlegm	58 (34.7)	54 (38.0)	^a^ 0.548
	Urine	47 (28.1)	46 (32.4)	^a^ 0.417
	Wound site	17 (10.2)	3 (2.1)	^a^ 0.004 **
	Other (pleural fluid, Peritoneum, etc.)	2 (1.2)	2 (1.4)	^b^ 1.000
Agent	Acinetobacter	10 (6)	10 (7)	^a^ 0.707
	*Kleasiella*	22 (13.2)	22 (15.5)	^a^ 0.561
	*Pseudomonas*	25 (15.0)	21 (14.8)	^a^ 0.964
	*E. coli*	57 (34.1)	39 (27.5)	^a^ 0.207
	*Enterococcus*	17 (10.2)	17 (12.0)	^a^ 0.616
	MRSA	4 (2.4)	6 (4.2)	^b^ 0.522
	MSSA	8 (4.8)	7 (4.9)	^a^ 0.955
	Candida	8 (4,8)	9 (6.3)	^a^ 0.552
	Other	17 (10.2)	16 (11.3)	^a^ 0.758

^a^ Pearson Chi-Square Test, ^b^ Fisher’s Exact Test, ** *p* < 0.01. MSSA: methicillin-sensitive Staphylococcus aureus, MRSA: Methicillin-Resistant Staphylococcus aureus, HT: Hypertension, DM: Diabetes Mellitus, CVD: Cerebrovascular disease, CAD: Coroner artery disease.

**Table 3:** Evaluation of Age and Aiyochemical Measurements According to Mortality.

		Total	28-Day Mortality		*p*
			Survival (*n* = 167)	Non-Survival (*n* = 142)	
Age	Mean ± Ss	74.78 ± 13.92	73.85 ± 14.21	75.87 ± 13.54	^c^ 0.189
	Median (Min–Max)	77 (27–98)	77 (27–98)	78 (32–98)	
APAPCHE II	Mean ± Ss	30.99 ± 6.43	28.75 ± 5.98	33.63 ± 5.94	^d^ 0.001 **
	Median (Min–Max)	30 (15–51)	28 (15–46)	33 (18–51)	
SOFA	Mean ± Ss	9.17 ± 2.93	8.20 ± 2.70	10.32 ± 2.77	^d^ 0.001 **
	Median (Min–Max)	9 (1–17)	8 (2–17)	10 (1–17)	
Lactate	Mean ± Ss	3.32 ± 2.54	2.89 ± 1.95	3.83 ± 3.01	^c^ 0.001 **
	Median (Min–Max)	2.4 (1.4–24)	2.3 (1.4–18)	2.8 (1.6–24)	
AST	Mean ± Ss	96.57 ± 181.19	76.58 ± 154.54	120.08 ± 206.31	^c^ 0.011 *
	Median (Min–Max)	39 (6–1572)	35 (8–1572)	46.5 (6–1185)	
ALT	Mean ± Ss	55.36 ± 114.46	46.49 ± 96.48	65.8 ± 132.12	^c^ 0.252
	Median (Min–Max)	19 (2–849)	18 (2–753)	20 (5–849)	
Total bilirubin	Mean ± Ss	1.60 ± 3.52	0.99 ± 1.53	2.30 ± 4.83	^c^ 0.001 **
	Median (Min–Max)	0.7 (0.1–29.6)	0.6 (0.1–14.4)	0.8 (0.2–29.6)	
Direct bilirubin	Mean ± Ss	1.14 ± 2.92	0.63 ± 1.32	1.74 ± 3.98	^c^ 0.001 **
	Median (Min–Max)	0.3 (0.1–22.4)	0.3 (0.1–12.1)	0.4 (0.1–22.4)	
Urea	Mean ± Ss	121.32 ± 76.9	110.57 ± 76.62	133.95 ± 75.55	^c^ 0.001 **
	Median (Min–Max)	103 (14–494)	90 (14–494)	117.5 (19–404)	
Creatinine	Mean ± Ss	2.65 ± 1.86	2.59 ± 2.04	2.73 ± 1.63	^c^ 0.087
	Median (Min–Max)	2.2 (0.3–10.8)	2 (0.3–10.8)	2.4 (0.3–7.7)	
FIB-4	Mean ± Ss	6.49 ± 7.89	4.83 ± 6.37	8.44 ± 9.02	^c^ 0.001 **
	Median (Min–Max)	3.8 (0.3–54.6)	3.3 (0.3–54.6)	5.3 (0.4–45)	

^c^ Mann–Whitney U Test; ^d^ Student’s *T* Test; * *p* < 0.05, ** *p* < 0.01. APAPCHEII: Acute Physiology And Chronic Health. Evaluation SOFA: Sequential Organ Failure Assessment, FIB-4: Fibrosis-4 Index.

**Table 4:** Diagnostic Screening Tests and ROC Curve Results for FIB-4 Measurement.

Diagnostic Scan							ROC Curve	
	Cut-Off	Sensitivity	Specificity	Positive Predictive Value	Negative Predictive Value	Area	95% Confidence Interval	*p*
FIB-4	4.9	4.92	4.25	4.46	4.95	0.672	0.612–0.732	0.001 **

ROC: Receiver Operating Characteristic, FIB-4: Fibrosis-4 Index; ** *p* < 0.01.

**Table 5:** Logistic regression results of factors affecting upgrade.

			95% C.I.ODDS	
*p*		ODDS	Lower	Upper
DM (+)	0.105	0.606	0.331	1.110
Septic shock (+)	0.528	0.810	0.421	1.558
Wound site infection	0.058	0.258	0.064	1.045
Lactate	0.519	1.043	0.918	1.184
AST	0.775	1.000	0.998	1.001
Total Bilirubin	0.680	0.872	0.456	1.669
Urea	0.695	0.999	0.995	1.003
APAPCHEII	0.000 **	1.101	1.008	1.156
SOFA	0.037 *	1.122	1.007	1.251
Direct Bilirubin	0.042 *	1.228	1.080	1.497
FIB-4 (≥4.9)	0.006 **	2.127	1.237	3.659

* *p* < 0.05, ** *p* < 0.01. APAPCHEII: Acute Physiology And Chronic Health Evaluation SOFA: Sequential Organ Failure Assessment FIB-4: Fibrosis Index-4.

## Data Availability

The data used in this study are available upon reasonable request.
